# Local and systemic inflammatory lipid profiling in a rat model of osteoarthritis with metabolic dysregulation

**DOI:** 10.1371/journal.pone.0196308

**Published:** 2018-04-23

**Authors:** H. M. de Visser, S. C. Mastbergen, S. Ravipati, P. M. J. Welsing, F. C. Pinto, F. P. J. G. Lafeber, V. Chapman, D. A. Barrett, H. Weinans

**Affiliations:** 1 Department of Orthopaedics, University Medical Center Utrecht, Utrecht, The Netherlands; 2 Department of Rheumatology & Clinical Immunology, University Medical Center Utrecht, Utrecht, The Netherlands; 3 School of Pharmacy (DAB, FCP) and School of Life Sciences (VC), University of Nottingham, Nottingham, United Kingdom; 4 Arthritis Research UK Pain Centre, School of Life Sciences, University of Nottingham, Nottingham, United Kindom; 5 Department of Biomechanical Engineering, Delft University of Technology, Delft, The Netherlands; Max Delbrueck Center for Molecular Medicine, GERMANY

## Abstract

**Objective:**

Bioactive oxidised lipids (oxylipins) are important signalling mediators, capable of modulating the inflammatory state of the joint and anticipated to be of importance in joint homeostasis and status of osteoarthritis. The aim of this study was to quantify oxylipin levels in plasma and synovial fluid from rats with experimentally induced osteoarthritis to investigate the potential role of oxylipins as a marker in the disease process of early osteoarthritis.

**Design:**

Forty rats were randomly allocated to a standard or high-fat diet group. After 12 weeks, local cartilage damage was induced in one knee joint in 14 rats of each diet group. The remaining 6 rats per group served as controls. At week 24, samples were collected. Oxylipin levels were quantified by liquid chromatography–mass spectrometry.

**Results:**

Overall, 31 lipid-derived inflammatory mediators were detected in fasted plasma and synovial fluid. Principal component analysis identified four distinct clusters associated with histopathological changes. Diet induced differences were evident for 13 individual plasma oxylipins, as well as 5,6-EET in synovial fluid. Surgical-model induced differences were evident for three oxylipins in synovial fluid (15-HETE, 8,9-DHET and 17R-ResolvinD1) with a different response in lipid concentrations for synovial fluid and plasma.

**Conclusions:**

We demonstrate the quantification of oxidised lipids in rat plasma and synovial fluid in a model of early experimental osteoarthritis. Oxylipins in the synovial fluid that were altered as consequence of the surgically induced osteoarthritis were not represented in the plasma. Our findings suggest differential roles of the oxylipins in the local versus peripheral compartment.

## Introduction

The presence of (low-grade) inflammation in osteoarthritis(OA) is well-known and is considered of relevance in the pathophysiological process of OA[1]. Many patients with OA have signs of mild inflammation such as local warmth, pain and joint effusion[2, 3]. Synovial inflammation can be present in early, as well as late, phases of OA, and is associated with synovial-related molecules released into biological fluids[4]. Previously, we demonstrated that systemic metabolic and subsequent inflammatory mediators, combined with a mild surgical trigger of local cartilage damage in the rat contribute to the progression of OA[5]. One of the characteristics of the induced metabolic dysregulation in this model is dyslipidaemia, which is also linked to clinical OA pathophysiology[6, 7]. In this model the progression of joint degeneration was driven mainly by the systemic and local inflammatory responses, as demonstrated by enhanced synovitis, osteophytosis and increased recruitment of macrophage lineage (CD68 expressing) cells[5]. Thus, this model mimics key aspects of the health status of human OA synovial joints, including increased inflammatory status and changes in synovial fluid lipid profiles [8, 9]. Indeed, there is evidence for altered lipid metabolism contributing to OA pathology via promotion of inflammation, apoptosis, and angiogenesis[10]. Oxidised lipids (oxylipins) are important signalling mediators capable of modulating the inflammatory state of a joint and might have an important role in the OA pathogenesis[1]. Polyunsaturated fatty acids are classified as n-3 (omega-3) or n-6 (omega-6).[11] Oxylipins can origin from linolenic acid (octadecanoids), a n-6 fatty acid, and arachidonic acid which is a product of its elongation/desaturation producing eicosanoids.[12–14] Another origin of oxylipins is n-3 unsaturated fatty acids synthesized from α-linolenic acid, including eicosapentaenoic- (EPA) and docosahexaenoic-acid (DHA).[12, 15] These omega-3 fatty acids have proven to be beneficial in modulating the inflammatory processes.[15, 16] Specifically, bioactive eicosanoid oxylipins, have a crucial role in modulating physiological processes in both homeostatic and inflammatory conditions[17–19]. Eicosanoids are 20-carbon fatty acid derivatives, produced from arachidonic acid[20]. The production of pro-inflammatory and/or anti-inflammatory eicosanoids, that include prostaglandins, thromboxanes, leukotrienes, and lipoxins, as well as other bioactive lipids, increases during inflammation[21–24]. During inflammation, eicosanoids regulate cytokine production, antibody formation, cell proliferation, migration, and antigen presentation but also control the tissue repair process[22]. Bioactive eicosanoid oxylipins are considered a quantitative readout relating to the inflammatory and oxidative stress status, and so may provide an early diagnostic and prognostic biomarker of disease[25]. However, due to the potent biological signalling activity of enzymatically oxidised lipids, the active mediators are short-lived in systemic circulation where they are actively metabolised prior to excretion[26]. Plasma levels of bioactive oxylipins as a representative of the OA situation might therefore not be the most suitable approach to study the lipid profile in the process of OA. The local lipid profile from joint tissues is more likely a better representative of the current OA status of the joint. As the metabolite concentrations in synovial fluid can directly reflect the joint homeostatic conditions that are related to biological processes of articular cartilage and other joint tissues, possibly already in the early stages of the disease[27, 28]. In humans with symptomatic knee OA changes in systemic levels of lipids have are associated with OA[29], and in end-stage OA patients, altered lipid levels and increased levels of pro-inflammatory cytokines have been detected in synovial fluid samples[30]. Potential alterations that may occur at onset or during early phases of OA may be more relevant to understand disease progression, before the joint is fully degenerated. At the moment, a validated biochemical biomarker to detect molecular events related to early disease activity in OA, either of systemic or local origin, is still lacking[31].

The aim of the current study was to identify the potential role of systemic and local inflammation in the OA disease process and to test if systemic oxylipin levels reflect the local status in synovial fluid. Therefore, we quantified the bioactive oxylipin levels in plasma and synovial fluid in an experimental rat model of early OA, with local cartilage damage in addition to a high-fat diet induced metabolic dysregulation.

## Methods

### Animal model

Forty Wistar rats (12 weeks old, male, Charles-River, Sulzfeld, Germany), housed two per cage in a 12:12 light-dark cycle, were randomly divided in two groups: twenty rats were fed a high-fat diet (HFD; 60% of the kcal contained fat: D12492i, USA) while the other rats received a standard diet (9% of the kcal contained fat: 801730, SDS, Essex, UK) with access to food pellets and tap water *ad libitum*. After 12 weeks, cartilage damage was induced, under general anesthesia, on the femoral condyles by placement of five grooves without damaging the underlying subchondral bone, in one knee joint according to the rat groove model[32] in 14 rats of each diet group. Analgesia (Buprenorphine) was provided until 24 hours after surgery and all animals were immediately allowed to move freely.The remaining 6 rats in each group served as control group without sham surgery for each diet. Sham surgery was not performed as previous work demonstrated no difference in synovial inflammation or cartilage degeneration 12 weeks after sham surgery compared to non-operated control joints[33]. The study design is based on our published methods combining a HF diet and the rat groove model of OA[5]. At endpoint rats were euthanized in their home cage using carbon dioxide and dead was confirmed by respiratory arrest together with fixed and dilated pupils. Joint degeneration was assessed as previously described using the OARSI histopathology score specifically for rats according the guidelines[34]. The total OARSI score is based upon the sum of the following sub sections: cartilage matrix loss width (0–2), cartilage degeneration (0–5), cartilage degeneration width (0–4), osteophytes (0–4), calcified cartilage and subchondral bone damage (0–5) and synovial membrane inflammation (0–4).The study was approved by the Utrecht University Medical Ethical Committee for animal studies (DEC 2013.III.12.086) and ARRIVE guidelines were fully complied.

### Sample collection

At week 24, all rats were fasted for 6 hours and subsequently blood was collected via the lateral tail vein. Blood samples were centrifuged at 3000 RCF for 15 minutes and plasma was stored at −80°C until analysed. Subsequently rats were euthanized by carbon dioxide and the synovial fluid of the experimental knee joints collected immediately afterwards. To collect the synovial fluid, first the skin of the hind paw was removed and the *M*.*quadriceps* was dissected following the quadriceps reversing approach[35]. With the knee in flexion an Ahlstrom 226 filtration paper of 3mm section (PerkinElmer, USA) was introduced in the knee joint for maximum absorption of the synovial fluid as previously described [36–38] preventing contact with surrounding tissues. Subsequently, the absorbed synovial fluid on the filter papers were placed in a 2ml Eppendorf tube and directly snap frozen and stored at -80°C upon analysis.

### Extraction of samples

Internal standards (10 μl of PGF2a-EA-d4 (2.49 μM), 10 μl of AA-d8 (1 μM), 10 μl of PGD2-d4 (1 μM), 10 μl of 15-HETE-d8 (7.6 μM) were added to each sample or blank sample (0.4 ml water), along with 2 μl of formic acid (98% v/v) and 5 μl of an antioxidant butylhydroxytoluene (BHT). Samples were homogenised in micro centrifuge tubes with the addition of 900 μl of ethanol, followed by a slow vortex stage (10 min) and centrifuged (13000 g, 10 min, 4°C). The supernatants were transferred to glass tubes and diluted by the addition of 3ml water. The diluted supernatants were loaded to the Strata-X polymeric SPE column (200mg/6 ml, Phenomenex, Macclesfield, UK) that had been preconditioned with 100% ethanol (2ml) and 25% ethanol (4 ml). The SPE cartridge then washed with distilled water (10 ml) and 25% ethanol (5 ml). The eicosanoids were eluted from the column with ethyl acetate containing 0.0002%BHT (5 ml) and were dried in centrifugal evaporator. The samples were reconstituted in 100% ethanol (100 μl) and 20 μl was injected for LC-MS/MS analysis.

### LC-MS/MS method

Concentrations of 34 oxylipins (see S1 Table) were quantified by liquid chromatography–mass spectrometry (LS-MS/MS) using a validated quantitative method based on that described by Wong et al.[21]. Lipids not included in [21] were measured using the following LC-MS/MS settings (precursor / product ions / collision energy): 17R resolvin D1 (375.3 / 141.0 / 22), 6-ketoPGF1a (369.1 / 162.8 / 36), resolvin D2 (375.3 / 175.0 / 30), 17-HDoHE (343.1 / 343.1 / 9). The HPLC system used was a Shimadzu series 10AD VP LC system (Shimadzu, Columbia, MD, USA) and the MS system used was an Applied Biosystem MDS SCIEX 4000 Q-Trap hybrid triple-quadrupole–linear ion trap mass spectrometer (Applied Biosystem, Foster City, CA, USA) equipped with an electrospray ionisation (ESI) interface. Quantification of the eicosanoids was calculated using fully extracted calibration standards for each of the analytes. Before quantification of lipid-derived inflammatory mediators of rat synovial fluid, blank filter papers were stained with equivalent concentrations of calibration standards (100pM, 500pM, 1nM, 2.5 nM, 5nM, 10nM) and were compared with actual standard calibration curves. Quantification was performed using Analyst 1.4.1. Identification of each compound in plasma samples was confirmed by LC retention times of each standard and precursor and product ion *m/z* ratios.

### Statistical analysis

First a principal component analysis (PCA) was performed on all analytes from synovial tissue and plasma together. Subsequently, data of all analytes were normalised and the z-scores were presented as mean with standard deviation. To test for the differences between the four different study groups for the selected clusters, a 1-way ANOVA with a Bonferroni correction was used. To evaluate the effect of HF diet and groove surgery on the normalised average data for each cluster of lipids an independent samples t-test was performed.

Potential associations of lipids within the selected clusters, formed by PCA, with the histological joint degeneration, as determined by the OARSI score[34], were determined with a linear regression analysis. The outcome is presented as regression coefficient (B) for linear regression with 95% confidence interval. In parallel, to relate the selected clusters (as formed by PCA) with the individual components of the histological OARSI-score; synovial membrane inflammation, osteophyte formation, and cartilage degeneration, a logistic regression analysis was used. Data are presented as odds ratios (OR) with 95% confidence intervals. All individual lipid data from serum and synovial fluid are separately presented as absolute mean value with standard deviation distinguished by the type of diet or the performed surgical procedure. To evaluate the effect of HF diet feeding and groove surgery on each individual lipid an independent samples t-test was performed. Finally, the correlation between the individual lipids and the histological OARSI-score was performed by a Pearson correlation (SPSS statistics 21, SPSS inc., Chicago, IL, USA). For all tests p values <0.05 were considered statistically significant.

## Results

### Association of systemic and local lipids with histological joint degeneration

**Fig 1:**
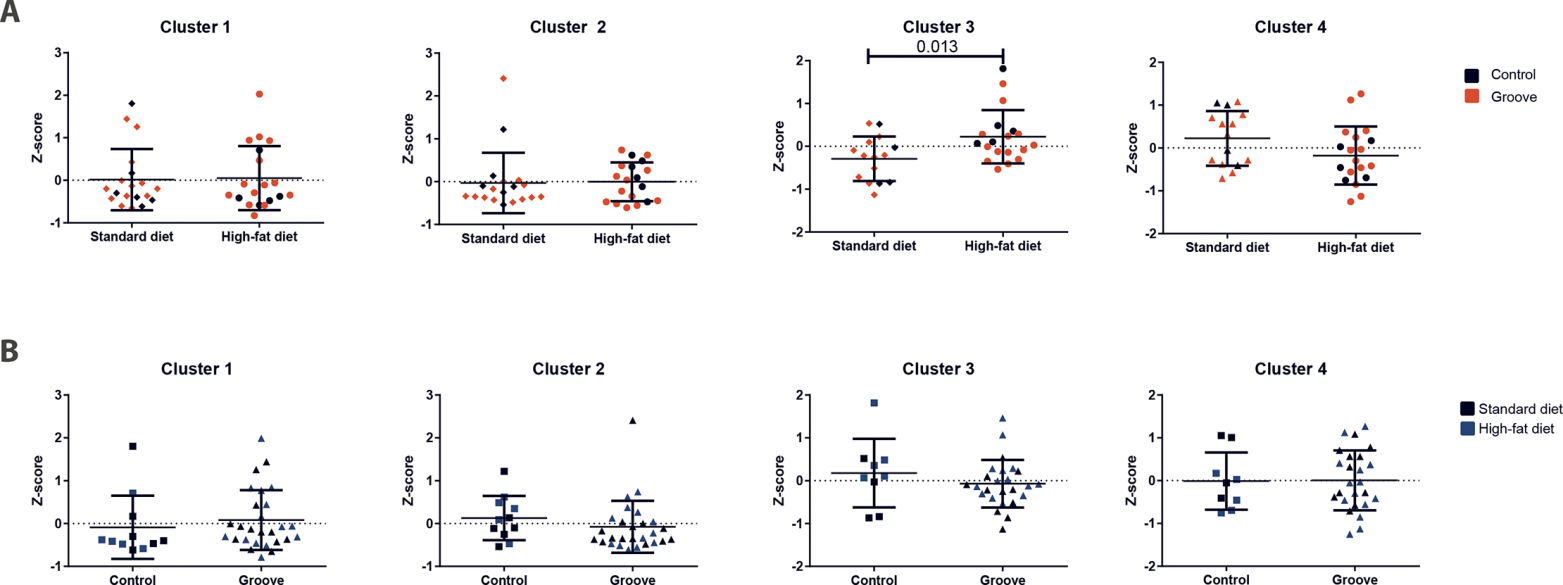
Overview of lipids within the selected clusters. Overview of normalised values for all lipids within the selected clusters for individual animals. The difference between the standard diet rats compared to the high-fat diet rats are presented above (**A**). The individual animals indicated by a red symbol are rats with groove surgery for each dietary group. The differences between rats with groove surgery compared to the non-operated rats are shown below (**B**). The blue symbols indicate rats on a high-fat diet. P-value was determined by the independent samples t-test.

Overall 31 of 34 oxylipins could be detected in rat fasted plasma and synovial fluid (obtained on filter papers). PCA identified four different clusters, ranging from 7–17 individual oxylipins (Table 1). The individual oxylipins within each cluster were strongly associated. The clusters differentiated clearly between lipids from local and systemic origin; cluster 1 and 2 only containing lipids from the synovial fluid and cluster 3 and 4 only lipids from plasma. When considering the effect of HF diet (with and without groove surgery), a statistical significant increase in averaged normalised lipid value of cluster 3 was observed, compared to the standard diet fed rats (with and without groove surgery; z-score of 0,22 ± 0,62 vs. -0,29 ± 0,52; p = 0.013, Fig 1A). All other clusters did not show a difference between the HF diet and the standard diet fed rats. Also, no differences were observed in rats with mechanical induced cartilage damage (grooves) compared to non-surgically damaged rats for all clusters (Fig 1B).

**Table 1:** Selected clusters of oxylipins as defined by the principal component analysis.

Cluster 1	Cluster 2	Cluster 3	Cluster 4
5-HETE (SF)	8,9-DHET (SF)	5-HETE (P)	8,9-DHET (P)
8-HETE (SF)	5-HPETE (SF)	8-HETE (P)	11,12-DHET (P)
9-HETE (SF)	12-HPETE(SF)	9-HETE (P)	5-HPETE (P)
11-HETE (SF)	9-HODE (SF)	11-HETE (P)	12-HPETE (P)
12-HETE (SF)	13-HODE(SF)	12-HETE (P)	9-HODE (P)
19-HETE (SF)	9-OxoODE (SF)	15-HETE (P)	13-HODE (P)
8,9-EET (SF)	13-OxoODE (SF)	16-HETE (P)	13-OxoODE (P)
11,12-EET (SF)	17-HDoHE (SF)	19-HETE (P)	17-HDoHE (P)
14,15-EET (SF)	AA (SF)	8,9-EET (P)	AA (P)
TXB2 (SF)		11,12-EET (P)	
PGD2 (SF)		14,15-EET (P)	
6-KetoPGF1a (SF)		TXB2 (P)	
ResolvinD1 (SF)		PGD2 (P)	
PGF2 (SF)			
ResolvinD117R (SF)			

Overview off all individual lipids within the selected clusters as defined by the principal component analysis. Two clusters contain solely oxylipins originating from the synovial fluid (SF) filter papers (Cluster 1 and 2), whereas the other two groups contain lipids originating from plasma samples (P; Cluster 3 and 4).

Looking at the association of the normalized lipid values within the different clusters for the total joint degeneration (independent of the four groups), as determined by the total OARSI histopathology score, using linear regression analysis, cluster 1 and cluster 3 showed a non-significant positive association with the total joint degeneration score. Indicating that increased histological joint degeneration is association with increased lipid concentrations, from local and systemic origin (Table 2). The other two clusters (cluster 2 and 4) showed a negative trend with the histological OARSI score (Table 2). When considering the association of the four clusters with the individual parameters of the histological OARSI score (logistic regression analysis), the association was in line with the total OARSI score (Table 2). A statistically significant positive association between systemic oxylipins in cluster 3 and the local synovial membrane inflammation (OR 54,78 [2.6–1170.9]; p = 0.010, Table 2) was found. The synovial fluid lipids in cluster 2 showed a statistically significant negative association with cartilage degeneration (OR 0.004 [0.0–0.5]; p = 0.024, Table 2).

**Table 2:** The outcome of logistic regression analysis.

	Total OARSI score	Synovial membrane inflammation	Cartilage degeneration	Osteofyt formation
Cluster 1 (Local)	B = 0,50 [-0,9–1,9]; p = 0,464	OR = 1,38 [0,3–5,6]; p = 0,650	OR = 2,33 [0,3–18,5]; p = 0,423	OR = 1,63 [0,4–6,7]; p = 0,498
Cluster 2 (Local)	B = -0,67 [-2,4–1,0]; p = 0,417	OR = 1,17 [0,2–6,1]; p = 0,855	**OR = 0,004 [0,0–0,5]; p = 0,024**	OR = 0,50 [0,1–4,2]; p = 0,521
Cluster 3 (Systemic)	B = 0,86 [-0,6–2,4]; p = 0,248	**OR = 54,78 [2,6–1170,9]; p = 0,010**	OR = 3,94 [0,5–30,3]; p = 0,188	OR = 1,88 [0,5–7,7]; p = 0,381
Cluster 4 (Systemic)	B = -1,1 [-2,5–0,3]; p = 0,124	OR = 0,24 [0,1–1,2]; p = 0,084	OR = 0,28 [0,1–1,7]; p = 0,170	OR = 0,35 [0,1–1,4]; p = 0,127

The outcome of logistic regression analysis from selected clusters in relation to selected individual histological parameters (Synovial membrane inflammation, Osteophyte formation and Cartilage degeneration) is shown. Data is presented as odds ratio (OR) with 95% confidence interval. In the right column outcome of linear regression analysis with histological joint degeneration score (total OARSI score) is shown. Data is presented as regression coefficient (B) with 95% confidence interval for the total OARSI score.

### Plasma and synovial fluid lipid profiles

**Fig 2:**
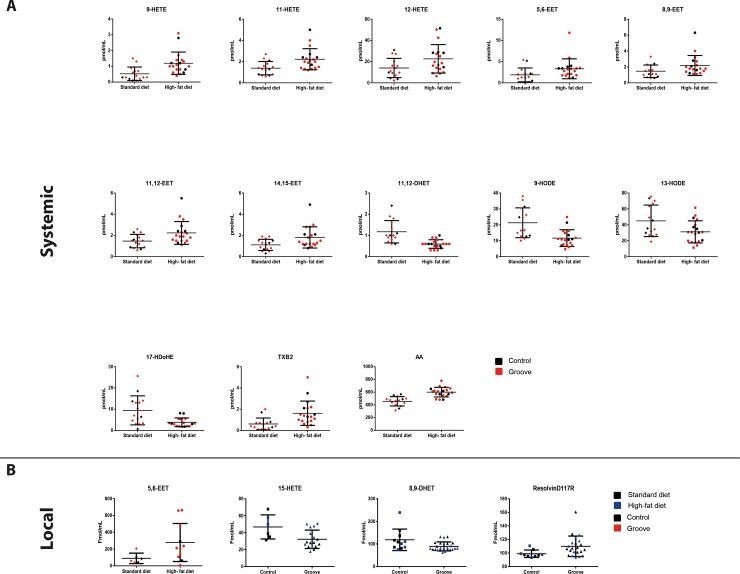
Overview of all statistical significant different individual lipids. Overview of all statistical significant different individual lipids originating from plasma (systemic, **A**) and synovial fluid (local, **B**), as determined by the independent samples t-test. Data is presented as an absolute value of the lipid for each individual animal. The individual animals indicated by a red symbol are rats with groove surgery for each dietary group and the blue symbols indicate rats on a high-fat diet.

**Fig 3:**
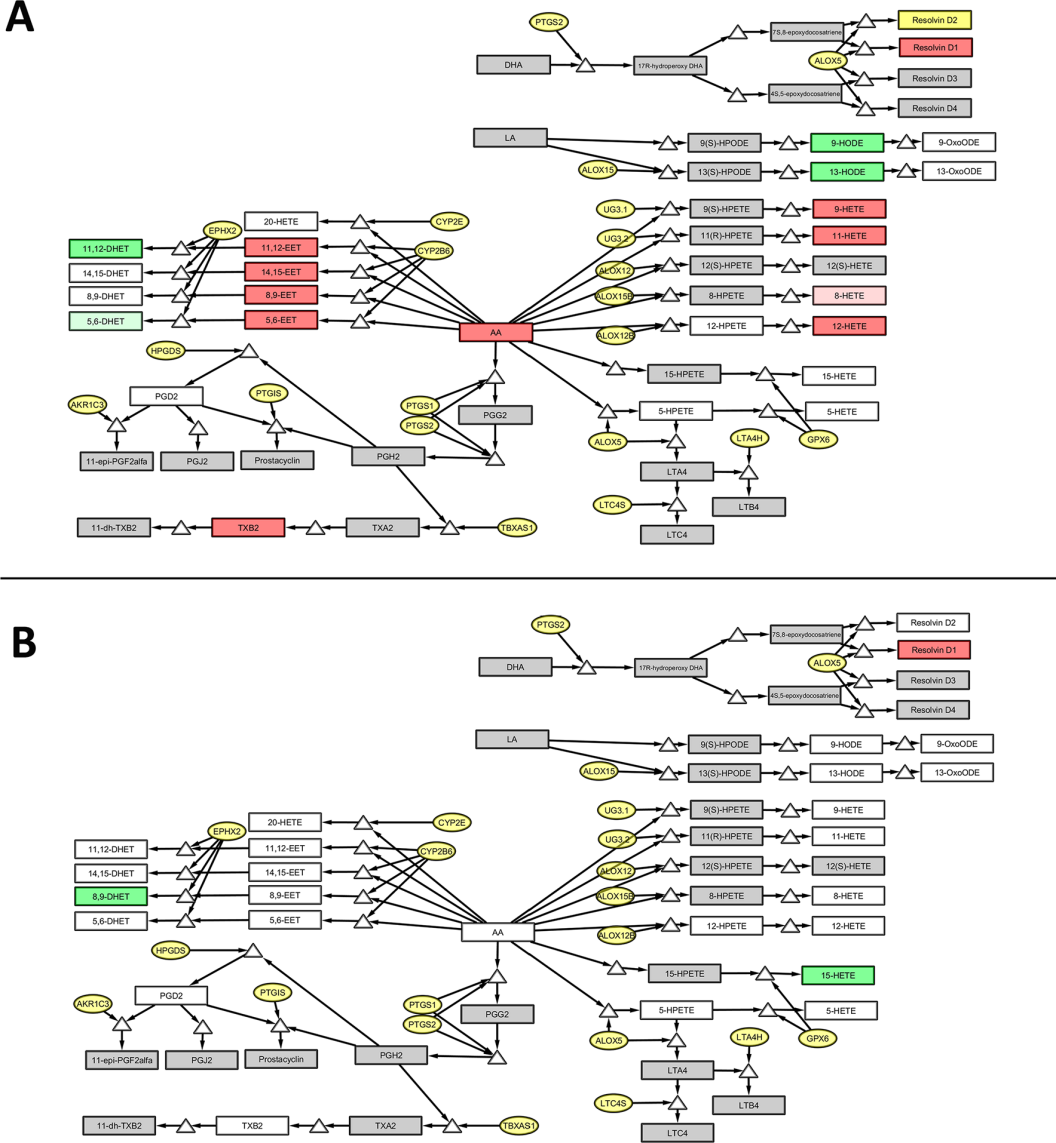
Representative pathway of all studied lipids. Pathways representing a selection of the most important studied oxylipins in plasma (A) and synovial fluid (B), mapped onto the main arachidonic acid (AA), docosahexaenoic acid (DHA) and linoleic acid (LA) metabolic pathways, corresponding to the observed changes in Fig 2. The green boxes represents statistical significant higher values in control situation compared to the HF diet (**A**) or groove surgery (**B**), and red boxes for statistical significant higher oxylipin values in the HF diet (**A**) or groove surgery (**B**) compared to their control situation.

Of the 31 detected lipids, 13 lipids present in the fasted plasma were significantly different between the HF diet and the standard diet group, independent for the performed groove surgery (9-HETE; p = 0.002, 11-HETE; p = 0.005, 12-HETE; p = 0.033, 5,6-EET; p = 0.044, 8,9-EET; p = 0.048, 11,12-EET; p = 0.015, 14,15-EET; p = 0.014, 11,12-DHET; p = 0.001, 9-HODE; p = 0.002, 13-HODE; p = 0.031, 17-HDoHE; p = 0.007, TXB2; p = 0.002 and AA; p = 0.0001; see Table 3 and Fig 2A). Whereas, only one lipid (5,6-EET; p = 0.023) present in the synovial fluid was higher in the HF diet group, compared to standard diet group, independent of groove surgery. Local cartilage damage following groove surgery had an effect on individual lipid values in the synovial fluid only, not fasted plasma, independent of diet. In total, levels of three oxylipins in the synovial fluid (15-HETE; p = 0.016, 8,9-DHET; p = 0.051 and ResolvinD117R; p = 0.006) were significantly different in the rats with groove surgery, compared to the non-surgically damaged rats (Table 3 and Fig 2B). Fig 3 shows the significantly changed oxylipins in plasma and synovial fluid mapped onto the main arachidonic acid (AA), docosahexaenoic acid (DHA) and linoleic acid (LA) metabolic pathways. The pattern of oxylipins changes, is shown to be linked with the activity of key enzymes such as cytochrome P450 (CYP), soluble epoxide hydrolyse (encoded by EPHX2 gene) and arachidonate 5-lipoxygenase (encoded by ALOX5 gene).

**Table 3:** Overview of all determined individual oxylipins.

		Systemic (pmol/mL)				Local (pmol/sample)		
Lipid	Standard	HF	Control	Groove	Standard	HF	Control	Groove
5-HETE	5,6 ± 2,5	6,9 ± 3,0	6,9 ± 3,7	6,1 ± 2,5	132 ± 51,1	174 ± 78,2	148 ± 61,4	154,6 ± 72,2
8-HETE	1,5 ± 0,8	2,2 ± 1,3	2,1 ± 1,8	1,8 ± 0,8	61,6 ± 30,1	90,4 ± 59,0	68,3 ± 33,4	79,1 ± 53,6
9-HETE	0,5 ± 0,4	1,2 ± 0,7	0,9 ± 0,8	0,9 ± 0,6	69,6 ± 37,0	68,0 ± 36,8	56,7 ± 36,9	74,1 ± 35,6
11-HETE	1,4 ± 0,6	2,2 ± 1,0	2,1 ± 1,3	1,8 ± 0,8	919 ± 862	1534 ± 1351	1279 ± 1198	1205 ± 1167
12-HETE	14,0 ± 9,1	22,6 ± 13,4	22,3 ± 15,5	17,6 ± 11,1	473 ± 444	1333 ± 1997	579 ± 618	1035 ± 1720
15-HETE	0,5 ± 0,3	0,7 ± 0,4	0,7 ± 0,4	0,6 ± 0,3	32,0 ± 12,4	40,3 ± 13,2	46,8 ± 14,2	32,2 ± 10,8
16-HETE	0,4 ± 0,2	0,4 ± 0,1	0,4 ± 0,1	0,4 ± 0,1	22,4 ± 17,3	30,5 ± 18,0	30,3 ± 22,9	24,7 ± 15,0
19-HETE	2,7 ± 1,3	2,1 ± 0,6	2,8 ± 1,4	2,2 ± 0,8	1081 ± 288	1059 ± 290	1032 ± 334	1085 ± 269
20-HETE	198 ± 183	285 ± 214	207 ± 128	246 ± 222	41,9 ± 30,6	34,1 ± 33,1	35,5 ± 38,4	38,2 ± 30,4
5,6-EET	1,9 ± 1,6	3,3 ± 2,4	2,6 ± 1,7	2,7 ± 2,3	89,3 ± 62,6	278 ± 226	118 ± 100	250 ± 227
8,9-EET	1,5 ± 0,8	2,2 ± 1,3	2,1 ± 1,7	1,8 ± 0,8	68,5 ± 34,2	98,0 ± 61,5	72,6 ± 41,5	87,6 ± 55,0
11,12-EET	1,5 ± 0,6	2,2 ± 1,1	2,2 ± 1,4	1,8 ± 0,7	884 ± 898	1554 ± 1431	1297± 1270	1187 ± 1231
14,15-EET	1,1 ± 0,5	1,8 ± 1,0	1,6 ± 1,4	1,5 ± 0,7	643 ± 542	1527 ± 2139	807 ± 680	1198 ± 1852
5,6-DHET	0,8 ± 0,4	0,6 ± 0,2	0,8 ± 0,3	0,7 ± 0,3	nd	nd	nd	nd
8,9-DHET	0,3 ± 0,1	0,3 ± 0,1	0,3 ± 0,1	0,3 ± 0,1	94,2 ± 34,2	96,4 ± 39,4	118,3 ± 47,5	85,6 ± 26,2
11,12-DHET	1,2 ± 0,5	0,6 ± 0,2	0,9 ± 0,6	0,8 ± 0,4	nd	nd	nd	nd
14,15-DHET	1,5 ± 0,6	1,2 ± 0,3	1,5 ± 0,6	1,3 ± 0,4	28,7 ± 19,7	38,6 ± 33,1	47,3 ± 34,4	26,3 ± 19,9
5-HPETE	3,1 ± 1,5	3,9 ± 2,6	3,2 ± 1,8	3,6 ± 2,3	136 ± 114	229 ± 195	207 ± 167	174 ± 167
12-HPETE	2,2 ± 1,3	3,0 ± 2,0	2,5 ± 1,8	2,7 ± 1,8	308 ± 107	428 ± 256	325 ± 139	174 ± 167
9-HODE	21,3 ± 9,4	11,6 ± 5,2	16,2 ± 7,9	15,8 ± 9,2	3,5 ± 3,5	3,2 ± 2,1	3,7 ± 3,1	3,2 ± 2,8
13-HODE	45,0 ± 19,8	31,2 ± 13,8	39,5 ± 15,2	36,4 ± 19,0	6,1 ± 6,3	5,7 ± 4,2	6,9 ± 5,9	5,5 ± 5,1
9-OxoODE	25,1 ± 11,3	23,4 ± 17,8	21,6 ± 10,9	25,0 ± 16,4	2755 ± 5202	1137 ± 964	1998 ± 1094	1849 ± 4383
13-OxoODE	34,1 ± 16,3	30,3 ± 23,0	29,9 ± 17,3	32,7 ± 21,3	2,8 ± 5,2	1,4 ± 1,2	2,3 ± 1,4	2,0 ± 4,4
17-HDoHE	9,5 ± 6,9	3,8 ± 2,1	7,0 ± 5,9	6,2 ± 5,5	2,9 ± 1,9	2,7 ± 1,4	2,9 ± 1,9	2,8 ± 1,5
AA	456 ± 73	600 ± 76	538 ± 91	536 ± 109	61,2 ± 28,2	84,4 ± 59,8	76,2 ± 34,8	71,4 ± 52,5
ResolvinD117R	65,5 ± 26,5	70,1 ± 12,8	65,1 ± 26,0	69,1 ± 18,2	97,7 ± 4,1	110,8 ± 17,1	98,9 ± 5,5	109,9 ± 15,1
TXB2	0,6 ± 0,6	2,1 ± 1,5	1,6 ± 1,0	1,0 ± 1,0	1,5 ± 2,5	3,2 ± 4,5	2,8 ± 3,3	2,2 ± 3,9
PGD2	0,3 ± 0,1	0,4 ± 0,4	0,4 ± 0,3	0,3 ± 0,3	1,4 ± 1,9	2,2 ± 2,1	2,1 ± 2,7	1,7 ± 1,7
6-KetoPGF1a	0,3 ± 0,2	0,2 ± 0,1	0,3 ± 0,1	0,3 ± 0,1	0,3 ± 0,1	0,3 ± 0,1	0,3 ± 0,2	0,3 ± 0,1
ResolvinD2	2,9 ± 2,5	2,2 ± 1,3	3,0 ± 2,0	2,4 ± 1,9	0,2 ± 0,1	0,2 ± 0,1	0,2 ± 0,1	0,2 ± 0,1
PGF2	nd	nd	nd	nd	1,4 ± 2,3	2,0 ± 1,4	2,4 ± 2,8	1,5 ± 1,4

Overview of all determined individual oxylipins for both plasma (systemic) and synovial fluid (local) samples of rats receiving high fat (HF; *n* = 20) compared to standard diet fed rats (Standard; *n* = 20) and the rats with groove surgery (Groove; *n* = 28) compared to rats without groove surgery (Control; *n* = 14). Data is presented as mean with the standard deviation of each individual lipid, nd = not detectable. The dark grey highlighted boxes indicates a statistical significant difference (p<0.05) and the light grey boxes indicates a trend compared to the control group (p<0.1) as tested by the independent samples t-test.

Of the individual lipids, there was a correlation with the histological outcome for four lipids in the plasma (9-HODE; r = -0.416, p = 0.014, 13-HODE; r = -0.426, p = 0.012, 17-HDoHE; r = -0.406, p = 0.019 and AA; r = 0540, p = 0.001). Two lipids in the synovial fluid were correlated with the histological joint degeneration (12-HETE; r = 0.330, p = 0.043 and 12-HPETE; r = 0.321, p = 0.05).

The diol/epoxy-ratios were also studied and the high-fat diet resulted in a statistically significant decreased in all diol/epoxy-ratios studied systemically in plasma samples (5,6-DHET/5,6-EET-ratio; p = 0.0049,8,9-DHET/8,9-EET-ratio; p = 0.0016, 11,12-DHET/11,12-EET-ratio; p<0.0001 and 14,15-DHET/14,15-EET-ratio; p<0.0001). When groove surgery is applied in high-fat diet fed rats also all diol/epoxy-ratios decreased compared to rats with groove surgery in addition to a standard diet (5,6; p = 0.0174, 8,9; p = 0.0298, 11,12; p<0.0001 and 14,15-; p = 0.0023). Looking at the local levels of diol/epoxy-ratios from synovial fluid samples, we didn’t see an effect of the high-fat diet compared to the standard diet in all levels. Also groove surgery in addition to a high-fat diet did not result in different diol/epoxy-ratios. In line with the individual oxylipins, a different expression was seen in the peripheral and local compartment.

## Discussion

The present study profiled oxylipin levels in both synovial fluid and plasma from a rat OA model, combining mechanically induced cartilage damage with a HF diet, using a highly sensitive LS-MS/MS method. Multiple clusters of oxylipins, as determined by PCA, were associated with histopathological changes by logistic regression analysis. Whereas, 4 local (5,6-EET, 15-HETE, 8,9-DHET and 17R-ResolvinD1) and 13 systemic oxylipins were clearly altered in this OA model as a result of groove surgery, HF diet feeding or a combination of both induced triggers. With distinct differences in synovial fluid and plasma concentrations of individual oxylipins, suggesting differential roles of the oxylipins in the local versus peripheral compartment.

Focusing on the individual lipids in the synovial fluid we observed a statistically significant decrease of 15-HETE levels in the synovial fluid of grooved knee joints compared to non-surgically damaged joints. 15-HETE is known to be secreted by adipocytes[39], but how this lipid is related in the process of OA is currently unknown. In mice, the absence of 15-HETE resulted in accelerated joint swelling and has an anti-inflammatory role[40]. On the other hand, increased levels of 15-HETE are present in knee joints of MIA induced rats[21], and a positive association of 15-HETE with incidence of human symptomatic knee OA is observed[29]. Besides 15-HETE, local changes were also observed for, 8,9-DHET, a pro-inflammatory lipid, 17R-Resolvin D1, a mediator of inflammatory responses, and 5,6-EET, which has a known role in pain mechanisms[41–43]. These altered lipid values were only observed in synovial fluid and not in plasma, except for 5,6-EET which is the only lipid increased in both the local and peripheral compartment. Moreover, the 13 plasma lipids that were sensitive to the HF diet in plasma were not reflected in the synovial fluid. This data suggest differential roles for oxylipins in the local versus peripheral compartment.

When looking at group level rather than looking at the individual lipid values, a strong positive association is detected between systemic oxylipins and the histological synovitis-score. This implies a direct effect of systemic circulating pro-inflammatory lipids on the local inflammatory state of the joint. On the other hand, the cluster with identical lipids from local origin did not show an association with knee joint synovitis. Another cluster of lipids originating from synovial fluid, showed a negative association with histological cartilage degeneration, indicative for a protective effect of these lipids on the articular cartilage. Previously we showed that osteophyte formation was an inflammatory driven process, in the selected model[5]. However, in the present study no association with osteophytes was observed in selected clusters of systemic and local lipids.

To better understand the complex inter-relationship between the different disease mechanisms involved in OA, animal models can help to elucidate the complex mechanistic aspects of OA[32, 44]. The advantage of using synovial fluid samples is that it is in direct contact with the tissues of the knee joint and likely contain more specific biomarkers that reflect the primary joint related degeneration pathways[45]. However, for humans the availability of synovial fluid from healthy but also early-OA patients is limited[28] and challenging due to the difficulty of defining early-OA[46]. Comparative lipidomic analysis of synovial fluid in a canine model of OA and human early-OA revealed that the lipid profiles of dogs often reflect those of humans[8]. Whether also small animal models of OA reflect the human disease with respect to lipid profiles, is currently unknown. In a model of HF diet induced obesity with destabilization of the medial meniscus in mice, an association was found between serum and synovial fluid lipid levels with histological OA and synovitis[47]. Although these data are in line with previous studies using a HF diet in rats with changed systemic lipid values[48, 49], disadvantage of destabilizing the meniscus is permanent joint instability and joint inflammation making the translation to the human OA situation questionable. This model could potentially be used to test medical interventions. Specifically the Cytochrome P450 system could be an interesting target to focus on, this system constitute a major metabolic pathway for arachidonic acid.[50] Besides, there is evidence that blocking of CYP enzymes with *N*-methylsulfonyl-6-(2-propargyloxyphenyl) hexanamide (MS-PPOH) represent a therapeutic target.[51] As prolonged MS-PPOH delivery result in attenuated effects in pulmonary hypertension[52], antidiuretic effects[53], and decreased coronary reactive hyperemia after ischemia due to inhibition of EETs synthesis.[54] This makes MS-PPOH a potential beneficial therapy for this model, where we can study the oxylipin profiles to better understand the metabolic changes associated with the inhibition of CYP epoxygenases.

During the initial phase of inflammatory responses in symptomatic knee OA, cyclooxygenase-2 is significantly up-regulated and acts on arachidonic acid to produce oxylipin mediators, specifically prostaglandins, prostacyclins, and thromboxanes[55, 56]. Oxylipins of both the cyclooxygenase and lipoxygenase pathways have been produced in sufficient quantities by joint tissues to be reflected in plasma in patients with symptomatic knee OA, indicating an increased arachidonic acid metabolism in OA[29]. Also local levels of endocannabinoid lipids in human synovial fluid and the infrapatellar fat pad in relation to OA have previously been reported[21]. In the current study increased plasma dihydroxyeicosatrienoic acids (DHETs) levels and decreased corresponding epoxyeicosatrienoic acids (EETs) levels were observed. EETs are very unstable metabolites, it's rapidly hydrolyzed by soluble epoxide hydrolase to the less biologically active but more stable metabolites DHETs and EETs might reflect the state of inflammation.[57] This indicates that the observed decreased plasma diol/epoxy-ratios might be involved in the inflammatory reaction as seen in this OA model.

Besides regulating inflammation, oxylipins are also important mediators of inflammatory pain[56]. Especially the role of resolvin receptors in pain behaviour have been studied[58]. Inhibitory effects of a precursor of resolvin D1, 17(R)-HDoHE, were observed on established OA pain in rats[58], which is corroborated in our study showing decreased levels of systemic 17(R)-HDoHE in rats with a HF diet. In our study, pain-related outcome measures were not performed and therefore further research needs to support this.

Local molecular biomarkers from the knee joint in small animals are limited by the small volume and difficult accessibility of the synovial joints and therefore often not taken into account[37, 59]. Often blood plasma samples are used as a representative of general inflammatory status with maybe some by-product that originates from the joint fluid. As such blood plasma oxylipins may be useful as biomarkers that can elucidate joint condition. The present study for the first time profiled local lipids originating from solely synovial fluid in rat knee joints. To access the synovial fluid, we selected the Whatman paper recovery method as previously designed for animals with small volumes of synovial fluid[36–38]. This specific and sensitive quantitative assessment method has the capacity to study pathway profiling of selected inflammatory related oxylipins, thereby providing a useful tool for the observation of biological differences and a readout for inflammation and oxidative stress in (experimental) early-OA. However, the statistical results have to be interpreted with caution as this experimental study had an exploratory nature and group size was not specifically designed for this research question. Irrespectively, specific changes in lipids related to inflammation as a consequence of a HF diet and induction of local cartilage damage by groove surgery could already be demonstrated and although the association between the selected histological outcome parameters and oxylipins do not necessarily reflect a causal relationship, they warrant further investigation of the role of the eicosanoid system in early OA mechanisms.

Here we present for the first time that it is possible to quantify (mainly eicosanoid) oxylipins in rat synovial fluid in an early experimental model of OA with local cartilage damage in addition to a HF diet induced metabolic dysregulation. It was demonstrated that both local and systemic bioactive oxylipins are responsive in early stages of the osteoarthritic process especially in the inflammatory responses involved and that local and systemic responses are not directly related. The HF diet induced metabolic dysregulation mainly influenced the systemic oxylipins of the fasted plasma. Whereas the mechanically induced cartilage damage with groove surgery had the most effect on the local oxylipins originating from the synovial fluid. Further understanding of the mechanisms by which the selected lipids play a role in the process of (early-)OA is necessary for its potential role as biomarker of disease.

## Supporting information

S1 Table Standards used in lipid analysis. Lipids in **A** were purchased from Cambridge Bioscience (Cambridge, UK). Lipids in **B** were all purchased from Biomol International (Exeter, UK). Lipids in **C** Were purchased from Cayman chemical (MI, USA). All stock solutions of each compound were diluted in ethanol. Serial dilutions of these were used for calibration.
(PDF)
