# Supplementary material for: Local and systemic inflammatory lipid profiling in a rat model of osteoarthritis with metabolic dysregulation
Source: PLoS One. 2018 Apr 23;13(4):e0196308. doi: 10.1371/journal.pone.0196308 (PMC5912715; doi:10.1371/journal.pone.0196308)
Supplement: S1 Table — Lipids in A were purchased from Cambridge Bioscience (Cambridge, UK). Lipids in B were all purchased from Biomol International (Exeter, UK). Lipids in C Were purchased from Cayman chemical (MI, USA). All stock solutions of each compound were diluted in ethanol. Serial dilutions of these were used for calibration. (PDF) [file pone.0196308.s001.pdf]

A

Prostaglandin D2 ethanolamide (PGD2-EA)
Prostaglandin F2α ethanolamide (PGF2α-EA)
5,6-dihydroxyeicosatrienoic acid (5,6-DHET)
8,9-dihydroxyeicosatrienoic acid (8,9-DHET)
11,12-dihydroxyeicosatrienoic acid (11,12-DHET)
14,15-dihydroxyeicosatrienoic acid (14,15-DHET)
9-hydroxyeicosatetraenoic acid (9-HETE)
11-hydroxyeicosatetraenoic acid (11-HETE)
12-hydroxyeicosatetraenoic acid (12-HETE)
15-hydroxyeicosatetraenoic acid (15-HETE)
16-hydroxyeicosatetraenoic acid (16-HETE)
19-hydroxyeicosatetraenoic acid (19-HETE)
20-hydroxyeicosatetraenoic acid (20-HETE)
Arachidonic acid (AA)
5-hydroperoxyeicosatetraenoic acid (5-HPETE)
9-hydroxyoctadecadienoic acid (9-HODE)
13-hydroxyoctadecadienoic acid (13-HODE)
9-oxooctadecadienoic acid (9-oxoODE)
13-oxooctadecadienoic acid (13-oxoODE)
Prostaglandin D2 (PGD2)
Prostaglandin E2 (PGE2)
Thromboxane-B2 (TXB2)

B

5-hydroxyeicosatetraenoic acid (5-HETE)
8-hydroxyeicosatetraenoic acid (8-HETE)
5,6-epoxyeicosatrienoic acid (5,6-EET)
8,9-epoxyeicosatrienoic acid (8,9-EET)
11,12-epoxyeicosatrienoic acid (11,12-EET)
14,15-epoxyeicosatrienoic acid (14,15-EET)
12-hydroperoxyeicosatetraenoic acid (12-HPETE)

C

Resolvin D1
Resolvin D2
17-hydroxy-4Z,7Z,10Z,13Z,15E,19Z-docosahexaenoic acid (17-HDoHE)
